# Characterization and genomic analysis of Exiguobacterium alkaliphilum B-3531D, an efficient crude oil degrading strain

**DOI:** 10.1016/j.btre.2021.e00678

**Published:** 2021-09-28

**Authors:** Yanina Delegan, Yulia Kocharovskaya, Alexander Bogun, Angelika Sizova, Viktor Solomentsev, Leila Iminova, Nikita Lyakhovchenko, Alina Zinovieva, Mikhail Goyanov, Inna Solyanikova

**Affiliations:** a Skryabin Institute of Biochemistry and Physiology of Microorganisms, Pushchino Scientific Center for Biological Research of Russian Academy of Sciences, Pushchino, Moscow Oblast, 142290, Russian Federation; b Pushchino State Institute of Natural Science, Pushchino, Moscow Oblast, 142290, Russian Federation; c State Research Center for Applied Microbiology and Biotechnology, Obolensk, 142279, Russian Federation; d Belgorod State University, Belgorod, 308015, Russian Federation

**Keywords:** Exiguobacterium, Genome assembly, Crude oil, Biodegradation

## Abstract

The aim of the work was to carry out the physiological, biochemical and genetic characterization of the *Exiguobacterium alkaliphilum* B-3531D strain. This strain is promising for use in the field of environmental biotechnology, since it has a pronounced ability to utilize crude oil and individual hydrocarbons in a wide temperature range. The genome of the strain was sequenced and completely assembled, it consists of a 2,903,369 bp circular chromosome and two circular plasmids, namely, pE73 (73,590 bp) and pE52 (52,125 bp). When cultivated in a mineral medium containing 2% of crude oil, the strain utilized 50% within 30 days of the experiment. In simulated seawater with the same oil content, the loss of hydrocarbons was 45% over the same period. For the first time we observed in an *E. alkaliphilum* strain the ability to efficiently utilize crude oil, including with an increased content of sodium chloride in the cultivation medium.

## Highlights

• B-3531D is the first *E. alkaliphilum* strain with fully assembled genome.
• It is the first *E. alkaliphilum* strain with the ability to utilize crude oil.
• Strain utilized 34.5% of crude oil for 14 days at 28 °C and a salinity of 11%.

## 1 Introduction

The genus *Exiguobacterium* was proposed by Collins et al. [1] for an expanding group of coryneform bacteria which encompasses aerobically growing, asporogenous, non-partially-acid-fast, irregularly shaped Gram-positive rods [2]. The *Exiguobacterium* genus, a sister clade of the *Bacillus* genus, has not been sufficiently studied as yet. The interest this genus attracts is due to the fact that many Exiguobacteria were isolated from environmental conditions which are stressful for most microorganisms. Representatives of Exiguobacteria were isolated from suboptimal habitats such as the Uttarakhand Himalayas [3], the Siberian permafrost environment [4], a glacial ice core sample in Greenland [5], the stem of potato in Austria [6], and hot springs in the Yellowstone National Park [7]. Representatives of the genus *Exiguobacterium* have sufficient metabolic flexibility to adapt to high or low temperatures (they are able to grow at temperatures ranging from -12 to + 55 °C [8]), high and low atmospheric pressure [9], the presence of heavy metals in the environment [10], [11], [12]. Many Exiguobacteria are acido- or alkaliphilic [13].

Currently, the genus *Exiguobacterium* includes 19 species, 17 of which (except for *E. chiriqhucha* and *E. himgiriensis*) have been validated. In 2017, Gutiérrez-Preciado et al. [9] have described the N139 strain isolated from an alpine lake in the Andes. Alpine lakes located at an altitude of 3000–6000 m above sea level are characterized by high ultraviolet (UV) radiation and salinity, broad temperature variations, low nutrient concentrations and high contents of metals and metalloids, mainly arsenic [14,15]. The authors have found that bacteria from the genus *Exiguobacterium* are one of the dominant taxa under these conditions [11,16,17]. Using the N139 strain as a case study, Gutiérrez-Preciado et al. have proposed a new species of *E. chiriqhucha* and described its genome. They have also proposed the species *Exiguobacterium himgiriensis* [18] described on the basis of the type strain K22–26T, but it has not been validated.

Some representatives of Exiguobacteria are highly efficient degraders of crude oil pollution, including that in seawater [19]. Cai et al. [20] have described the strain N4–1P, a psychrophilic facultative anaerobic bacterium isolated from shoreline sediment samples contaminated with petroleum hydrocarbons in Newfoundland, Canada, using *n*-hexadecane or diesel as the sole carbon source. This was the first *Exiguobacterium* strain capable of using hydrocarbons as the sole carbon source and producing surfactants to stabilize oil-water emulsions [21,22]. Chen et al. (2017) reported about *Exiguobacterium* sp. isolated from oil-contaminated seawater with high degradation ability against crude oil. As part of the microbial community, represented by five strains, this bacterium decomposed up to 75% of oil in 7 days, while individually – 50.5% during the same period [23].

As of 2021, the GenBank database contains the genomes of 164 representatives of the genus *Exiguobacterium*. Of these, about 100 are not identified to the species level. The biotechnological potential of Exiguobacteria attracts the attention of researchers to the study of the genetic organization of these strains. For example, there is information on the genomes of three *E. acetylicum* strains, one of which is a complete assembly. Among the biotechnologically significant properties of *E. acetylicum* bacteria is the ability to express numerous plant growth promotion attributes, such as phosphate solubilization, indole acetic acid (IAA), siderophore and hydrogen cyanide (HCN) production [3], as well as many important enzymes, including lipase [24]. However, the currently available information on the genomes of different species of Exiguobacteria is rather limited.

Despite the fact that bacteria of this genus are characterized by a relatively small (less than 4 million base pairs) genome in comparison with other actinobacteria, nonetheless, such properties as the ability to biodegrade various pollutants, growth under suboptimal conditions, phyto-stimulation and halotolerance are characteristic of all Exiguobacteria. However, the information concerning the molecular biological characteristics of this genus is very limited so far.

The aim of the work was to carry out the physiological, biochemical and genetic characterization of the *Exiguobacterium* B-3531D strain. This strain is promising for use in the field of environmental biotechnology, since it has a pronounced ability to utilize crude oil and individual hydrocarbons in a wide temperature range. During the study, the B-3531D strain was completely sequenced and identified to species. This is the first complete assembly of a representative of the *E. alkaliphilum* species known to date.

## 2 Materials and methods

### 2.1 Microorganism isolation and culturing

The crude oil used as the carbon source had the following properties: density - 860 kg/m^3^, sulfur – 0.6%, resins and asphaltenes – about 5%, aromatic hydrocarbons – no more than 10%, viscosity – 14 mPa•*sec*. Lysogeny broth (LB) liquid medium [25] consisted of 10 g/L tryptone, 5 g/L yeast extract, and 5 g/L NaCl (in distilled water). Evans mineral medium [26] contained the following components (per 1 liter of distilled water): 8.71 g of K_2_HPO_4_, 1 ml of 5 M NH_4_Cl, 1 ml of 0.1 M Na_2_SO_4_, 1 ml of 62 mM MgCl_2_, 1 ml of 1 mM CaCl_2_, 1 ml of 5 μM (NH_4_)_6_Mo_7_O_24_•4H_2_O; 1 ml of trace elements, pH 7.0. The trace element solution consisted of the following components (in 1% HCl): 0.41 g/L ZnO; 5.4 g/L FeCl_2_•6H_2_O; 2.0 g/L MnCl_2_•4H_2_O; 0.17 g/L CuCl_2_• 2H_2_O; 0.48 g/L CoCl_2_•6H_2_O; 0.06 g/L H_3_BO_3_.

A sample of petroleum-contaminated soil was collected in the city of Yaroslavl (Russia). A weighed portion of 1 g of the sample was placed in 30 ml of Evans medium and thoroughly resuspended for 20 min. After waiting 5 min for soil particles to settle, 5 ml of the supernatant were transferred to 95 ml of sterile Evans medium with 2% (w/w) crude oil to obtain an enrichment culture. The cultivation was carried out for 7 days at a temperature of 30 °C and 180 rpm.

To obtain single bacterial colonies, a series of standard tenfold dilutions of the enrichment culture was carried out. 0.5 ml of the enrichment culture was resuspended in 4.5 ml of phosphate buffer, receiving the first dilution. 0.5 ml from the tube with the first dilution was resuspended in 4.5 ml of a new portion of phosphate buffer. The procedure was repeated sequentially until the 6th dilution was obtained. Then, 100 μl of the last dilution was plated onto a Petri dish with agarized Evans medium supplemented with 2% crude oil as the only source of carbon and energy. Single colonies were streaked three times using an inoculation loop onto the same medium to obtain pure cultures.

The B-3531D strain was tentatively identified as *Exiguobacterium* sp. by sequencing the 16S rRNA gene. Universal primers 27f/1492r [27] were used for PCR amplification of the 16S rRNA genes. The PCR thermal cycling consisted of an initial denaturation at 95 °C for 10 min, followed by 35 cycles at 94 °C for 45 s, 56 °C for 45 s, and 72 °C for 90 s, plus the final step at 72 °C for 10 min. Amplification was performed using a GeneAmp PCR System 2400. All amplification products were verified by electrophoresis on 1% agarose gel stained with ethidium bromide. The products were sequenced using an Applied Biosystems 3130 × 1 sequencer (ABI, USA) and the BigDye v.3.1 sequencing kit. The obtained sequences were subjected to BLAST homology search (http://www.ncbi.nlm.nih.gov/BLAST). We used the Clustal algorithm for sequence alignment and the MEGA7 software for constructing a phylogenetic tree [28]. All the sequences of the type strains for comparison with the sequences of our strains were taken from the GenBank NCBI database.

### 2.2 Genome sequencing, assembly and annotation

Genomic DNA was isolated from the biomass of a fresh culture (a colony) of *Exiguobacterium* sp. strain B-3531D grown on LB agar using the QIAamp DNA Mini Kit (cat. # 51,304; Qiagen). Sequencing was performed on a MinION sequencer with an R9.4.1 flow cell (Oxford Nanopore Technologies [ONT]) using the facilities of the State Research Center for Applied Microbiology and Biotechnology (SRCAMB, Obolensk, Russia). A library was prepared using the Rapid Barcoding Kit (cat. # SQK-RBK00). The Guppy version 3.2.4 software was used for base calling, which yielded a total of 2341.6 Mb distributed in 339,037 reads with a score *Q* > 10.

In addition, the same DNA sample was sequenced using an Illumina MiSeq platform with the MiSeq Reagent Kit v3 (cat. # MS-102–3003; 2  ×  300 bp). A paired-end library was prepared using the NEBNext® Ultra™ DNA Library Prep Kit. Quality control of Illumina reads was carried out through FastQC (http://www.bioinformatics.babraham.ac.uk/projects/fastqc). The Illumina and Nanopore reads were used for hybrid assembly with the SPAdes version 3.15.2 [29]. The Nanopore reads were assembled into contigs using the Flye assembler version 2.6 [30]. The SPAdes contigs were then combined into replicons using Flye data as reference. The Illumina reads were used to correct Nanopore errors using the Bowtie2 version 2.3.5.1 [31] and Pilon version 1.23 [32] software. Default settings were used for all software. Circularization of the ends of replicons (chromosomes and plasmids) was confirmed by overlapping ends, as well as by visualization in the Tablet program [33].

The data were submitted to the GenBank database under the accession numbers: BioProject – PRJNA721848, BioSample – SAMN18740301, GenBank – NZ_CP073101.1-NZ_CP073103.1.

The transfer RNAs (tRNAs) and ribosomal RNAs (rRNAs) of the B-3531-D strain were identified using the tRNAscan-SE [34] and RNAmmer (v1.2, http://www.cbs.dtu.dk/services/RNAmmer/) software, respectively. The assembled genome was annotated using Prokka [35] and RAST [36]. The functions of some proteins were checked manually using BLAST. The phylogenetic tree was constructed by the neighbor‐joining method using the REALPHY service [37]. Genome sequences of *Exiguobacterium* strains required for constructing the phylogenetic tree were taken from the WGS database (https://www.ncbi.nlm.nih.gov/Traces/wgs/?view=wgs). The circular maps were made using DNAPlotter [38].

ANI value was calculated using the EzBioCloud service [39]. DDH was calculated using the Genome-to-Genome Distance Calculator 2.1 service [40]. COG functional annotation was carried out using WebMGA [41]. We also used the Kyoto Encyclopedia of Genes and Genomes (KEGG) [42] to perform functional annotation using the blastp module.

### 2.3 Determination of temperature and pH limits of strain survival

To determine the optimal and limiting values of temperature and pH for the strain, 500 μl of a cell culture suspension equal to 0.1 OD (prepared using a Microscan Turbidity Meter, Siemens, USA) were inoculated into a liquid nutrient medium (composition: 30 g peptone, tap water to 1 L) in duplicate. To determine the optimal growth temperature, the inoculations were incubated in the temperature range from 4 °C to 50 °C. Every 4 h, optical density (OD) was measured at a wavelength λ=600 nm using a Genesys 20 spectrophotometer (Thermo Fisher Scientific, USA). To determine the growth properties of the culture at different pH and NaCl concentrations, the inoculations were incubated at the identified temperature optimum.

### 2.4 Determination of extent of crude oil degradation

The *E. alkaliphilum* strain B-3531D was cultivated in flasks with 50 mL of model seawater (Evans medium with/without sodium and calcium chlorides, 11% salinity) and crude oil (200 mg/L) for 30 days at 28 °C. The total content of oil hydrocarbons was measured by IR spectrometry after 14 and 28 days of experiment. After the cultivation, the residual oil was extracted with carbon tetrachloride (1:1). The content of hydrocarbons in the extract was determined with an AN-2 petroleum product analyzer (Russia). Sterile Evans medium with crude oil (200 mg/L) was used as a control. The crude oil concentration in liquid samples was calculated using the formula:

$$X=\frac{C\times V_{1}\times \eta }{V},\;\left[mg.L-1\right]$$

where C is the concentration of crude oil in the eluate determined from the instrument's readings or the calibration dependence, mg/L; Vi is the volume of the eluate, L; V is the volume of the water sample, l; η is the degree of eluate dilution. In the absence of dilution, η = 1.

## 3 Results

### 3.1 Sequencing and genome analysis of exiguobacterium sp. strain B-3531D

**Fig. 1:**
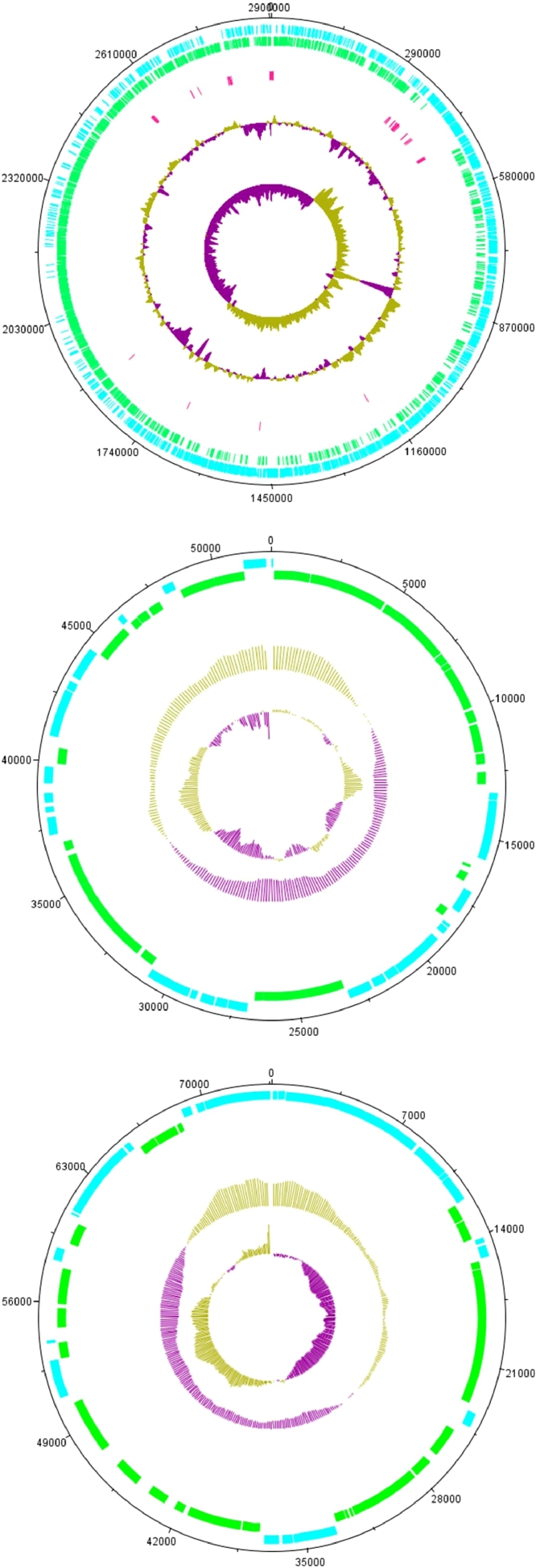
Circular maps of chromosome (a) of Exiguobacterium sp. B-3531D and two plasmids, pE52 (b) and pE73 (c). Outer tracks represent predicted CDS of the forward (cyan) and reverse (green) strands. Pink tracks represent RNAs. Two inner tracks display the GC plot and GC skew.

The genome of the strain was sequenced and completely assembled. The *Exiguobacterium* sp. B-3531D genome consists of a 2903,369 bp circular chromosome (GC content: 53.1%) and two circular plasmids, namely, pE73 and pE52. The pE73 size is 73,590 bp. (GC content: 42.4%), the pE52 size is 52,125 bp (GC content: 44.7%). The chromosome contains 3022 coding sequences, 9 rRNA clusters (5S, 16S, and 23S), 4 ncRNAs, and 66 tRNAs (Fig. 1a, b, c).

**Fig. 2:**
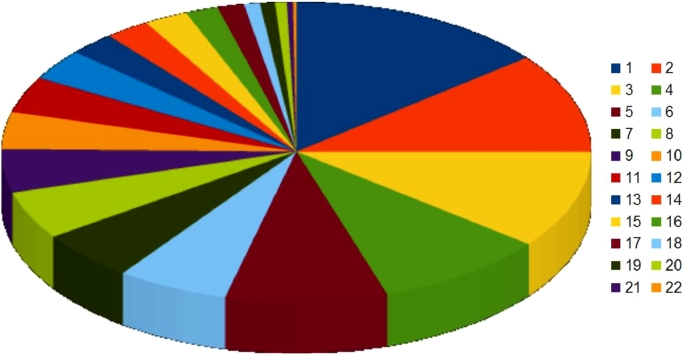
KEGG function classification of the strain Exiguobacterium sp. B-3531D. 1 – Carbohydrate metabolism, 2 – Protein families: signaling and cellular processes, 3 – Genetic Information Processing, 4 – Environmental information processing, 5 – Unclassified: metabolism, 6 – Amino acid metabolism, 7 – Unclassified, 8 – Metabolism of cofactors and vitamins, 9 – Nucleotide metabolism, 10 – Unclassified: genetic information processing, 11 – Cellular processes, 12 – Lipid metabolism, 13 – Protein families: metabolism, 14 – Energy metabolism, 15 – Unclassified: signaling and cellular processes, 16 – Glycan biosynthesis and metabolism, 17 – Metabolism of other amino acids, 18 – Metabolism of terpenoids and polyketides, 19 – Organismal systems, 20 – Biodegradation of xenobiotics, 21 – Biosynthesis of other secondary metabolites.

Out of the 3022 CDS found, 1719 (55.9%) were functionally annotated (Fig. 2). These results suggest that the *Exiguobacterium* sp. B-3531D strain has efficient lipid, carbohydrate and amino acid transport as well as metabolism.

### 3.2 Determination of taxonomic position of B-3531D strain

**Fig. 3:**
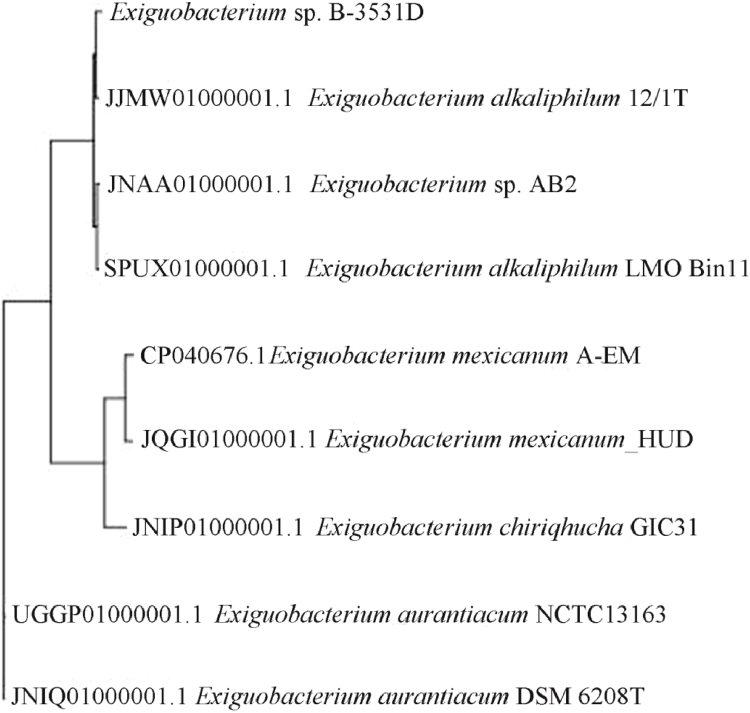
Neighbor‐joining phylogenetic tree of *Exiguobacterium* based on whole‐genome alignment.

To identify the strain, we used an integrated approach that included: 1) comparison of the chromosome sequences of the B-3531D strain with similar sequences of the closest related strains (Fig. 3), 2) calculation of the ANI parameter values, and 3) calculation of the DDH parameter values (Table 1).

**Table 1:** ANI and DDH values of Exiguobacterium sp. B-3531 and relative strains.

GenBank accession #	Strain name	ANI value,%	DDH value,%
JJMW01000001.1	*Exiguobacterium alkaliphilum* 12/1T	97.50	87.60
SPUX01000001.1	*Exiguobacterium alkaliphilum* isolate LMO Bin11	97.48	71.20
JNIQ01000001.1	*Exiguobacterium aurantiacum* DSM 6208T	87.67	81.30
UGGP01000001.1	*Exiguobacterium aurantiacum* strain NCTC13163	87.96	81.30
JNIP01000001.1	*Exiguobacterium chiriqhucha* strain GIC31	83.38	58.50
CP040676.1	*Exiguobacterium mexicanum* strain A-EM	83.01	54.70
JQGI01000001.1	*Exiguobacterium mexicanum*_HUD	82.98	48.40
JNAA01000001.1	*Exiguobacterium* sp. AB2	96.60	86.10
CP030931.1	*Exiguobacterium acetylicum*AMCC 101217	69.99	13.60
LFQN00000000.1	*Exiguobacterium acetylicum*ZBG2	69.64	13.50
JNIR00000000.1	*Exiguobacterium acetylicum* DSM 20416T	70.21	13.40

Among the strains taken for the analysis were type strains of the species *E. alkaliphilum, E. aurantiacum, E. acetylicum.*

The closest relative of the B-3531D strain is the *Exiguobacterium alkaliphilum* 12/1T strain, a type strain of the *E. alkaliphilum* species. The ANI and DDH values obtained by comparing the strains exceed the required threshold for species identification. Thus, in the future, we define the strain B-3531D as belonging to the species *E. alkaliphilum*.

### 3.3 Search for genes involved in hydrocarbon catabolism in strain B-3531D

When conducting the functional annotation of the B-3531D strain, we found certain genes involved in the degradation of benzoate, chloroalkanes and chloroalkenes, xylenes, ethylbenzene, and naphthalene. Previously, Edlund and Jansson [43] have noted the presence of the *phn*Ac gene (phenanthrene dioxygenase) in the phenanthrene-degrading *Exiguobacterium oxidotolerans* AE3 strain. The search for the *phn*Ac gene sequence taken from the *Exiguobacterium oxidotolerans* AE3 strain in the genome of our strain and related to it *Exiguobacterium alkaliphilum* 12/1T strain gave no results.

Since the genus *Exiguobacterium*, like the genus *Geobacillus* (many representatives of which are extremophilic degraders of alkanes), belongs to the phylum Firmicutes, the class Bacillus, we conducted a search for genes related to the genes *lad*A, *lad*B (*Geobacillus* genes involved in the destruction of alkanes) in the genome of the strain B-3531D. We did not find any related genes in the genome of our strain. We also did not find sequences related to the *alm*A gene involved in the catabolism of long-chain alkanes in extremophilic strains, as well as the *alk*B and cytochrome P450 alkane hydroxylase genes of bacilli. Sun et al. [44] have also noted that the genome of the *Exiguobacterium* strain which they studied does not contain the *phe*N (phenol hydroxylase), *alk*B, P450, and *alm*A genes.

Genes related to the genes of the naphthalene cluster of *Bacillus* and *Geobacillus* were also not found in the strain B-3531D and related *Exiguobacterium* strains. The naphthalene 1,2-dioxygenase gene (JQGI01000459.1) was found in the *Exiguobacterium mexicanum* strain HUD, but other *Exiguobacteria*, including our strain, do not have genes related to it. Therefore, it can be assumed that *Exiguobacteria* (including strain B-3531D) use an alternative mechanism for the catabolism of aromatic compounds in oil, rather than the naphthalene operon characteristic of bacilli.

We identified the catechol 2,3-dioxygenase gene in our strain and established that the sequence of this gene is widespread in the genomes of various species of Exiguobacteria, and its sequence is practically unchanged. However, we can only assume that this gene is involved in PAH catabolism by the B-3531D strain, since no other genes of the PAH catabolism pathway were found either in the vicinity of this gene or in the genome at all.

### 3.4 Physiological limits of strain survival

Exiguobacteria typically grow under conditions that are stressful for most widespread soil bacteria. The study of the physiological limits of survival of the strain allowed us to understand in which climatic zones its use will be efficient for the purposes of the biological remediation of petroleum-contaminated territories and waterbodies. We found that the strain has a rather wide range of survival, which is typical for a representative of Exiguobacteria (Table 2).

**Table 2:** Limit and optimum values of temperature, pH and salinity of the environment at which the growth of strain B-3531D in a mineral environment with oil as the only source of carbon and energy is possible.

	Limiting range	Optimum
Temperature, °С	10–42	28–30
рН	6–11	9
NaCl content in medium, g/L	0–150	10

For comparison, the optimum temperature range for the growth of *E. mexicanum* is between 30 °C and 40 °C, the optimum pH is ∼7 and the optimum NaCl concentration is 3% [45].

### 3.5 Properties of strain B-3531D as degrader of petroleum hydrocarbons

Under conditions of the experiment, the strain showed a reasonably good activity for crude oil degradation in a model saline solution. Within 14 days, the decline in the content of oil products comprised 34.5% under conditions of 11% salinity (data do not include abiotic decline). We also found that in the absence of salt in the model medium, the strain utilized 50% of the crude oil within 30 days of the experiment. The obtained results indicate that the strain can be used as a monoculture or as part of a consortium for the remediation of oil-contaminated soils and aquatic ecosystems.

## 4 Discussion

A certain degree of extremophilia is generally characteristic of representatives of the genus *Exiguobacterium*. The ability of representatives of this genus to grow under conditions that are stressful for most known microorganisms has attracted the attention of researchers around the world. The organization of genomes and the genetic determinants responsible for the extremophilia of Exiguobacteria and their metabolic flexibility are the subject of intensive studies. The genomes of some psychrophilic [46,47] and thermophilic [48] *Exiguobacteria* have been sequenced and assembled.

The GenBank database contains more than 100 sequenced genomes of Exiguobacteria belonging to different species. Most of the genomes have the draft status, and only a few have been fully assembled (Table 3).

**Table 3:** Completely assembled genomes of Exiguobacterium strains.

Strain name	Species	Genome size, Mb	Presence of plasmids (Y/N)
ZWU0009	*Exiguobacterium* sp.	3.25	N
U13–1	*Exiguobacterium* sp.	3.21	N
MH3	*Exiguobacterium* sp.	3.16	N
Helios	*Exiguobacterium* sp.	3.15	Y (100 kb)
AT1b	*Exiguobacterium* sp.	3.00	N
255–15	*Exiguobacterium sibiricum*	3.04	Y (4.8 kb, 1.7 kb)
B7	*Exiguobacterium antarcticum*	2.82	N
AMCC 101,217	*Exiguobacterium acetylicum*	3.16	N
A-EM	*Exiguobacterium mexicanum*	2.69	Y (68.7 kb, 4.5 kb, 4.2 kb)
SSD5 *	*Exiguobacterium profundum*	4.26	N

* nearly complete.

In this article, we for the first time report the complete assembly of a strain belonging to the *E. alkaliphilum* species. The available information on the genomes of Exiguobacteria allows us to conclude that representatives of this genus are characterized by small genomes which are sometimes accompanied by plasmids. The plasmids in the strains can range from a very small size with only a few reading frames to a much larger size, such as those in the *Exiguobacterium* sp. strain Helios or *E. mexicanum* strain A-EM.

Little information is available about the plasmids of Exiguobacteria. Ponder et al. [49] have published the sequences of the genome of the *Exiguobacterium sibiricum* strain 255–15 and its two plasmids, pEXIG01 (4885 bp) and pEXIG02 (1765 bp). The strain has been isolated from a 2–3 million year old permafrost core in Siberia, Russia, and it can survive and grow rapidly at low temperatures. Jakubauskas et al. [50] have described a 4563 bp pEspA plasmid and a 38,945 bp pEspB plasmid from the *Exiguobacterium arabatu*m sp. nov. strain RFL1109. The pEspB plasmid has been predicted to have 3 functional regions: the putative region of conjugative transfer, the region involved in the replication and maintenance of the plasmid, and the region responsible for the transposition of the IS21 family-like transposable elements.

During the annotation of the pE52 plasmid of our strain, we found some genes involved in sugar catabolism and elements of the Type III restriction-modification system. We did not find any catabolic genes or antibiotic resistance genes on either plasmid. The functions of both plasmids of the strain are not yet clear.

### 4.1 Search for genes presumably responsible for PGPR properties of B-3531D strain

Exiguobacteria are widely reported to be bacteria that have a positive effect on plant growth and development (PGPB). This effect is achieved through the synthesis of hormones that stimulate plant growth, nitrogen fixation, solubilization of nutrients, such as phosphorus. The positive effect is provided by mechanisms such as the synthesis of siderophores, chitinase activity, production of antibiotics and cyanides [51]. In addition to biodegrading properties, the B-3531D strain is likely to possess PGPR properties. In the genome of the strain, we found putative genes encoding a regulon for high affinity phosphate uptake, carbon starvation, cold shock and heat shock proteins. In the genome of the strain, we also identified genes involved in metabolism of siderophores and iron. Similar genes have earlier been reported in other works devoted to Exiguobacteria [52,53].

The gene cluster responsible for the phosphate uptake, as well as the genes involved in siderophore biosynthesis, are widespread in various species of Exiguobacteria. The results of studying the physiological properties of the B-3531D strain suggest that these genes are active and perform their functions of phosphorus solubilization and siderophore biosynthesis.

These results are consistent with those obtained by Tang et al. [52] who worked with the *Exiguobacterium* sp. strain MH3. The complete genome sequencing of *Exiguobacterium* strain MH3 isolated from the rhizosphere of *Lemna minor* have revealed the presence of many genes associated with stress tolerance, including putative genes encoding a regulon for high-affinity phosphate uptake, carbon starvation, oxidative stress, detoxification, and cold shock and heat shock proteins. Genes involved in auxin biosynthesis and metabolism of siderophores and iron have also been identified in the genome of this bacterium [52].

### 4.2 Potential application of B-3531D strain in environmental biotechnology field

The strain B-3531D is capable of degrading crude oil. Over a period of 30, 45 and 60 days, the content of total hydrocarbons in the mineral medium decreased by 50, 54 and 60%, respectively. This indicates that the strain B-3531D, like other Exiguobacteria, is inherent in the ability to degrade hydrocarbons. The B-3531D strain was shown to be capable of growth in the presence of 11% sodium chloride in the medium. In the presence of salt, the degradation efficiency decreased slightly. The decline in crude oil content was 45% and 52% in 30 and 48 days, respectively. Moreover, most of the hydrocarbons, up to 38%, were decomposed by the strain in the first two weeks. A decrease in the rate of biodegradation has also been shown for other microbial strains. This is due to the initial degradation of light oil fractions [23]. As shown earlier [18], strains of different species of Exiguobacteria (*E. himgiriensis* K22–26T, *E. aurantiacum* MTCC 6414T, *E. aquaticum* MTCC 10958T, *E. mexicanum* MTCC 7759T, *E. aestuarii* MTCC 7750T, *E. profundum* MTCC 10851T) are able to grow in the presence of 9% sodium chloride, so halotolerance can be considered a property of the genus. At the same time, for the B-3531D strain, this is precisely halotolerance, not halophilia; therefore, the strain can be used for remediation of ground and aquatic ecosystems in the presence of salt up to 11%.

The ability of Exiguobacteria to catabolize hydrocarbons has been reported many times [44,54]. Mohanty and Mukherji [19] have observed the ability of an *Exiguobacterium aurantiacum* strain to utilize diesel fuel as the only source of carbon and energy. The strain was also able to grow on alkanes in the C9-C26 range and on pristane. Erofeevskaya [55] has noted the ability of an *E. mexicanum* strain to degrade crude oil. By the 7th day of cultivation, the strain utilized 6.38% crude oil at +4 °С, 49.76% at +20 °С, 51.9% at +30 °С, and 57.78% at +37 °С. The factors influencing the efficiency of crude oil degradation include temperature, pH, salinity, initial oil concentration and age of pollution [23, 56]. *Exiguobacteria* sp. isolated from seawater, as part of a bacterial consortium, was able to degrade crude oil with greater efficiency when the temperature rises to 30 °C, salt - up to 30 percentiles, with pH values close to neutral. Immobilization of cells also had a positive effect on biodegradation processes [23]. As noted by Sutton et al., 2013, during the degradation experiment, a shift in the composition of oil pollution was observed in the direction of decreasing the relative amount of low molecular weight compounds, C10 - C12 and C12-C16 and an increase in the relative content of C20 - C24 and C24 - C40 fractions. With regard to aromatic hydrocarbons, there is an increase in the relative amounts of polycyclic compounds [23].

As for the representatives of *E. alkaliphilum*, their properties as oil degraders have not been previously reported. All information on the use of *E. alkaliphilum* in the field of environmental biotechnology concerns its ability to affect alkaline wastewater [57,58]. A type *E. alkaliphilum* strain has been isolated from wastewater with pH 12; it has been shown to be capable of neutralizing it to pH 7.5. Kulshreshtha et al. [58] have noted that neutralization of highly alkaline wastewater without the addition of any external carbon source highlights the potential use of the isolate as an alternative to the conventional acid neutralization methods for treatment of alkaline wastewater.

## 5 Conclusion

In this work, we for the first time report a complete assembly of the genome of a strain of the species *Exiguobacterium alkaliphilum*. All previously published genomes of representatives of this species have been assembled only to the contig level.

Moreover, we for the first time observed in an *E. alkaliphilum* strain the ability to efficiently utilize crude oil, including with an increased content of sodium chloride in the cultivation medium. The B-3531D strain utilized 34% of crude oil within 14 days at a salinity of 11%. Thus, we believe that the strain can be used for remediation of ground and aquatic ecosystems, including saline ones.

## Funding

All works except genomic sequencing were financially supported by 10.13039/501100002261Russian Foundation for Basic Research (Project No: 19–54–80,003).

Genome Sequencing was supported by the 10.13039/501100004569Ministry of Science and Higher Education of the Russian Federation (agreement number 075–15–2019–1671)

## Declaration of Competing Interest

Let me inform you that all authors (Yanina Delegan, Yulia Kocharovskaya, Alexander Bogun, Angelika Sizova, Viktor Solomentsev, Leila Iminova, Nikita Lyakhovchenko, Alina Zinovieva, Mikhail Goyanov, Inna Solyanikova) declare that they have no known competing financial interests or personal relationships that could have appeared to influence the work reported in this paper.
